# Activation of β1 integrins and caveolin-1 by TF/FVIIa promotes IGF-1R signaling and cell survival

**DOI:** 10.1007/s10495-020-01611-7

**Published:** 2020-05-27

**Authors:** Mikael Åberg, Desirée Edén, Agneta Siegbahn

**Affiliations:** Department of Medical Sciences, Clinical Chemistry and Science for Life Laboratory, Uppsala University Hospital, Entr. 61 3rd floor, 751 85 Uppsala, Sweden

**Keywords:** Apoptosis, Tissue factor, IGF-1R, Integrins, Caveolin-1

## Abstract

The tissue factor/coagulation factor VIIa (TF/FVIIa) complex induces transactivation of the IGF-1 receptor (IGF-1R) in a number of different cell types. The mechanism is largely unknown. The transactivation leads to protection from apoptosis and nuclear translocation of the IGF-1R. The aim of this study was to clarify the signaling pathway between TF and IGF-1R after FVIIa treatment with PC3 and DU145 prostate or MDA-MB-231 breast cancer cells as model systems. Protein interactions, levels, and phosphorylations were assessed by proximity ligation assay or flow cytometry in intact cells and by western blot on cell lysates. The transactivation of the IGF-1R was found dependent on TF/FVIIa-induced activation of β1-integrins. A series of experiments led to the conclusion that the caveolae protein caveolin-1 prevented IGF-1R activation in resting cells via its scaffolding domain. TF/FVIIa/β1-integrins terminated this inhibition by activation of Src family kinases and subsequent phosphorylation of caveolin-1 on tyrosine 14. This phosphorylation was not seen after treatment with PAR1 or PAR2 agonists. Consequently, the protective effect of FVIIa against apoptosis induced by the death receptor agonist TRAIL and the de novo synthesis of cyclin D1 induced by nuclear IGF-1R accumulation were both significantly reduced by down-regulation of β1-integrins or overexpression of the caveolin-1 scaffolding domain. In conclusion, we present a plausible mechanism for the interplay between TF and IGF-1R involving FVIIa, β1-integrins, Src family proteins, and caveolin-1. Our results increase the knowledge of diseases associated with TF and IGF-1R overexpression in general but specifically of TF-mediated signaling with focus on cell survival.

**Electronic supplementary material:**

The online version of this article (10.1007/s10495-020-01611-7) contains supplementary material, which is available to authorized users.

## Introduction

Tissue factor (TF) is a 47 kDa transmembrane glycoprotein and the initiator of the humoral coagulation. TF binds and activates its natural ligand coagulation factor VII (FVII) into FVIIa, an event that leads to activation of factor Xa (FXa) and thrombin and subsequently fibrin formation. Extravascular expression of TF provides a hemostatic envelope for protection if a vessel ruptures, whereas the intravascular expression normally is kept very low to avoid excessive clot formation [1]. Both in vitro and in vivo data consequently support a role for aberrant expression of TF in diseases associated with thrombosis such as cardiovascular disorders, diabetes, and cancer [2–4]. The TF/FVIIa complex also activates intracellular signaling pathways in a number of different cell types [5–9]. This implicates important roles for TF in different diseases uncoupled from its role in coagulation, which is exemplified by the high levels of TF found on many types of cancer cells where the signaling abilities of TF facilitates metastasis and spreading of tumors [10–12]. Today it is known that TF signaling can be transduced over the cell surface membrane in several ways. For instance, proteolytic activation by FVIIa is of importance for activating the G-protein-coupled protease-activated receptors (PAR) 1 and 2 [13], whereas a more direct physical interaction with TF/FVIIa seems significant for activation of certain β1-integrin (ITGβ1) complexes [14, 15]. TF also has a short intracellular domain containing three serines as potential phosphorylation sites [16].

One of the biological consequences of TF signaling is suppression of apoptosis. A protective role of the TF/FVIIa complex was first described in serum starved baby hamster kidney cells and Chinese hamster ovary (CHO) cells and later also in Adr-MCF7 and MDA-MB-231 breast cancer cells [17]. The reduction in caspase-3 activation was dependent on the proteolytic activity of FVIIa and the PI3-kinase/AKT signaling pathway, but independent of FXa and thrombin. Serum depletion from the MDA-MB-231 cells also activated caspase-8, the initiator caspase in the extrinsic pathway [18]. Similar to caspase-3, caspase-8 activation was sensitive to TF/FVIIa-dependent signaling. TNF-related apoptosis-inducing ligand (TRAIL) is expressed in immune cells of healthy individuals and induces apoptosis in a variety of cell types through binding to the DR4/DR5 death receptors [19–21]. A rapid up-regulation of both caspase-8 and -3 was recorded after TRAIL-receptor activation in both MDA-MB-231 breast and PC3 prostate cancer cells which was reduced after treatment with FVIIa [18].

The insulin-like growth factor 1 receptor (IGF-1R) is a transmembrane receptor tyrosine kinase (RTK) involved in regulating apoptosis, proliferation, differentiation, and motility. Altered IGF-1R signaling contributes to several pathological conditions including cancer [22]. Activation of the IGF-1R, either directly by its ligand IGF-1 or indirectly by other proteins through so called transactivation [23], results in phosphorylation of its intracellular β-subunits. We have previously recorded a dose-dependent transactivation of the IGF-1R in several principally different cell types: human breast cancer cells and human aortic smooth muscle cells with constitutive expressions of TF, human monocytes primed to express TF, and porcine aortic endothelial cells transfected with human TF, this after treatment with fully functional FVIIa but not after treatment with an active-site inhibited FVIIa [16, 24]. Inhibition or removal of IGF-1R blocked the FVIIa-mediated phosphorylation of AKT as well as the protection against TRAIL-induced apoptosis in cancer cells [24]. Treatment with FVIIa furthermore induced a translocation of a SUMOylated IGF-1R to the nuclei where it acted as a transcription enhancer and induced mRNA production. By using siRNA, receptor blocking antibodies and peptide agonists, PAR1 and PAR2 was ruled out as transducers of the survival signals directly downstream of TF/FVIIa [18, 24]. In a similar approach, we used porcine cells transfected with human TF with a modified cytoplasmic domain to show that TF did not have to be phosphorylated to transactivate IGF-1R [24]. In conclusion, it is today not known how the anti-apoptotic signal is transduced between TF/FVIIa and IGF-1R and the elucidation of this mechanism is therefore the subject of this study.

Both TF and IGF-1R have been associated with domains termed caveolae [25–27]. These are stable invaginations of the cell surface plasma membrane with a high content of proteins and act as signaling nodes. Caveolin-1 (Cav1) is the principal protein of the caveolae and has the ability to bind and regulate the activity of signaling proteins via an area between residues 82–101 termed the caveolin scaffolding domain [28, 29]. A conserved caveolin scaffolding domain binding motif has been identified in several RTKs including the IGF-1R [30].

We now demonstrate that TF/FVIIa-dependent ITGβ1 activation results in Cav1 Tyr14 phosphorylation by Src-family kinases. By interfering with ITGβ1, Src, or Cav1 activation we eliminated both the ability of TF/FVIIa to rescue cancer cells from TRAIL-induced apoptosis and the ability to induce IGF-1R-dependent protein production.

## Materials and methods

### Reagents

Cav1 scaffolding domain peptide and control peptide [31] were purchased from Merck Millipore USA. The Src-family inhibitors SU6656 and PP2, PAR1-activating peptide (SFLLRN), PAR2-activating peptide (SLIGKV), geranyl–geranyl pyrophosphate (GGPP), and simvastatin were bought from Sigma-Aldrich Sweden AB. Simvastatin was activated prior to the experiments by alkaline hydrolysis of the lactone ring according to the manufacturer’s protocol. Recombinant human FVIIa was a kind gift from Professor LC Petersen, NovoNordisk A/S, Denmark. The following antibodies were purchased: rabbit IGF-1Rβ, Cav1 rabbit, pSrc family Tyr416, Caspase-3, Cyclin-D1, β-actin, and pAbl Tyr245 from Cell Signaling Technology, USA; phosphotyrosine rabbit, HDAC-2 from Abcam, UK; Cav1 mouse from Santa Cruz Biotechnology, USA; Abl, activated β1 integrin (HUTS-4), and phosphotyrosine 4G10 Platinum from Merck Millipore; pCaveolin-1 Tyr14 from BD Transduction Lab, FITC-labeled activated β1 integrin from Bio-Rad, USA; FITC-labeled IgG from Dako Cytomation, USA; WB secondary antibodies IRDye 680CW (mouse and rabbit) and IRDye 800CW (mouse and rabbit) from LI-COR Biosciences, UK.

### Cell lines

PC3 and DU145 prostate cancer cells and MDA-MB-231 breast cancer cells were purchased from American Type Culture Collection (USA) and were grown in RPMI1640 (Gibco/Invitrogen) supplemented with 10% fetal bovine serum (FBS), 2 mM L-glutamine, 100 U/ml penicillin, and 100 µg/ml streptomycin in a humidified chamber at 37 °C, 5% CO2. All cell lines have previously been verified for their expression of TF [32].

### RNA interference

Silencer® Select Validated siRNA toward Cav1, ITGβ1, TF and scramble RNA (10 nM, Ambion/Life Technologies, USA) were transfected into the PC3 cells using Lipofectamine® RNAiMAX Transfection Reagent (Life Technologies). The reduction in expression levels (S-fig S1) and the functional experiments were assessed 72 h post transfection.

### Real-time quantitative PCR

Real-time quantitative PCR analyses of Cav1, GAPDH, ITGβ1, and β2 microglobulin (Assay-on-demand, Applied Biosystems, USA) were performed on cDNA originating from oligoDT (Invitrogen) converted total RNA extracted using Trizol® (Invitrogen). The samples were run and analyzed on an AbiPrism 7500 (Applied Biosystems).

### In situ proximity ligation assay

The proximity of antibodies targeting antigens of interest was monitored with the Duolink In situ assay (Sigma-Aldrich). Blocking solution, antibody dilution and washing buffers were supplied by the manufacturer. Briefly, PC3 cells were grown on chamber slides and then treated as indicated in the figures. The cells were then fixed in 2% paraformaldehyde, permeabilized in 0.2% TX-100 and blocked. Primary antibodies in optimized concentrations were added as indicated in the figures prior to the incubation with secondary oligonucleotide-conjugated PLA probes. If in close proximity (< 40 nm) and in the presence of a ligase, the PLA probes hybridized. Amplification solution, consisting of nucleotides and fluorescently labeled oligonucleotides, was added together with Polymerase to generate a rolling-circle amplification (RCA), resulting in an RCA product to which the fluorescently labeled oligonucleotides could hybridize. The localized amplification reaction increases the sensitivity by a 1000-fold and gives a lower limit of detection in the femtomolar range [33]. This gives a considerable increase in antibody/protein co-localization resolution also when compared to traditional double-label indirect immune fluorescence microscopy [34]. The nuclei and cytoskeleton (Fig. 4a only) were counterstained with DAPI and phalloidin respectively, and Z-stack images were captured using an AxioImager M2 fluorescence microscope at 20X magnification (Carl Zeiss AB, Sweden). The images were then analyzed using Blob Finder software (https://www.cb.uu.se/~amin/BlobFinder/). The images in the figures are magnifications from maximum-intensity projections over the entire image volume.

### Preparation of membrane and nuclear protein fractions

PC3 cells (0.75 × 10^6^ / ml) were either left untreated or were preincubated for 3 h with 5 nM with a Cav1 scaffolding domain membrane permeable peptide, and then stimulated with 10 nM or 100 nM FVIIa for 30 min. The cells were then lyzed and the membrane, nuclear soluble and chromatin-bound cellular fractions were separated using a Subcellular Fractionation Kit according to the manufacturer’s instructions (Thermo Scientific/Pierce Biotech, USA). The fractions were analyzed for the expression of IGF-1R and HDAC-2 by western blot (WB).

### Western blot

After being treated as indicated in the figures, the PC3 cells were lyzed in 2% SDS sample buffer supplemented with 5% beta-mercaptoethanol. The proteins were then separated by SDS-PAGE and transferred onto Immobilon-FL PVDF membranes (Merck Millipore). The membranes were blocked in Odyssey blocking buffer (Licor) and left overnight at 4 °C in blocking buffer containing the primary antibodies. The membranes were then washed in TBS 0.01% Tween-20 and incubated with secondary antibodies conjugated to IR-Dyes 680 and 800 for 60 min (Licor). The membranes were finally scanned and the bound proteins were visualized with the Odyssey Infrared Imaging System (Licor) and quantified using Odyssey V3.0 software.

### Flow cytometry

The PC3 cells were incubated with or without 10 nM FVIIa for 1, 5, 15, and 20 min and permeabilized with 0.2% Triton-X-100 in PBS for 15 min at room temperature. The cells were then washed in PBS with 0.5% FBS and incubated with FITC-labeled antibodies against the active conformation of ITGβ1 or IgG (isotype control). The protein expression was then analyzed in a Cytomics FC500 Flow Cytometer and the MFI values calculated using Kaluza Analysis Software (both Beckman Coulter Inc., USA).

### Statistics

Statistical values were calculated using Graph Pad Prism 8.0 (GraphPad software, Inc., USA) and the bars indicate mean + SEM. Unpaired Students t-test was used to evaluate the significance between two groups. For comparison of three or more groups, one-way ANOVA with Dunnett’s post-hoc calculation was applied. P-values ≤ 0.05 were considered statistically significant.

## Results

### The transactivation of IGF-1R by TF/FVIIa is conveyed by β1 integrins

**Fig.1:**
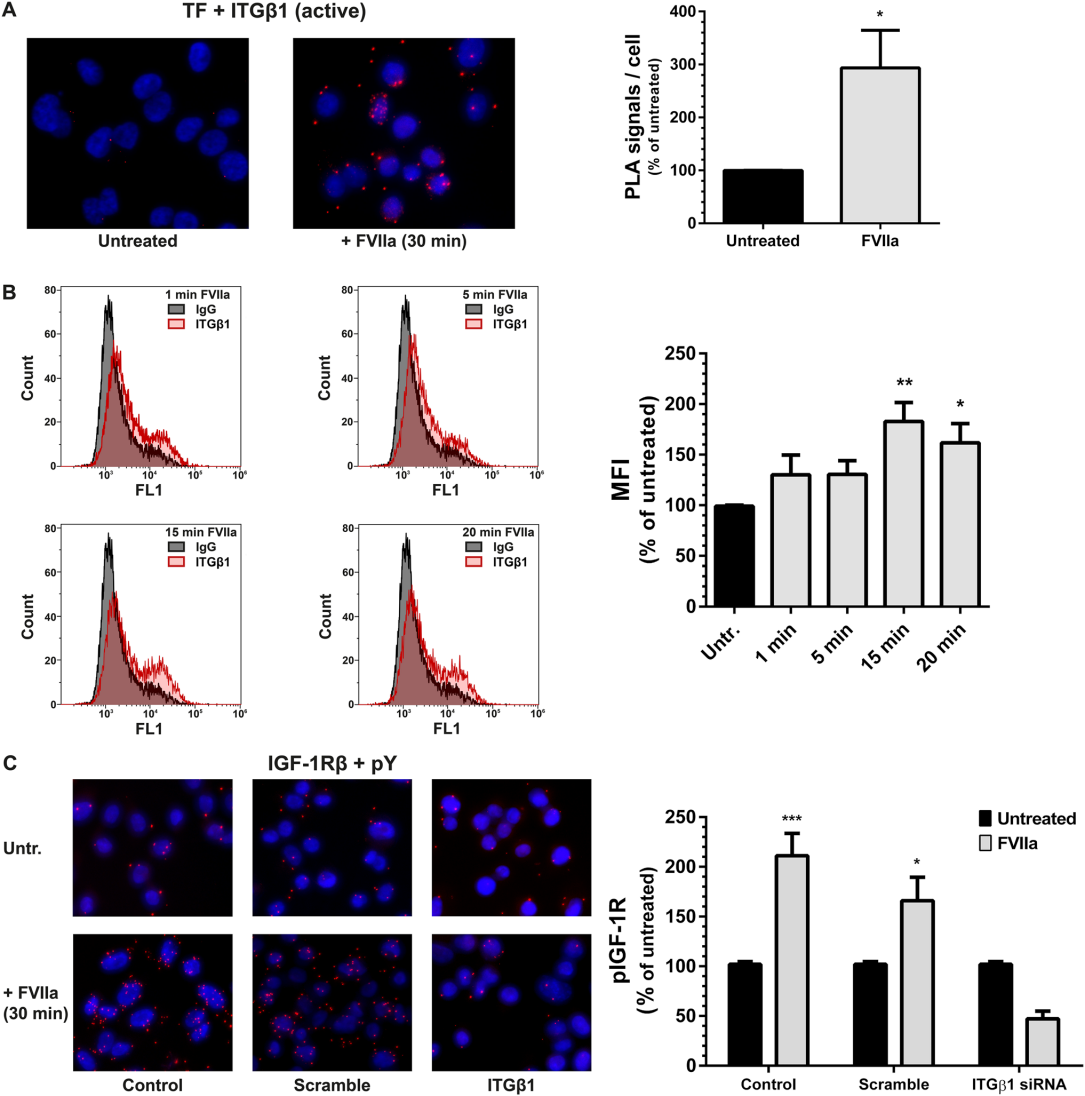
The transactivation of IGF-1R by TF/FVIIa is conveyed by β1-integrins **a** PC3 cells were treated with 100 nM FVIIa for 30 min, and the co-localization of TF and activated ITGβ1 was determined by PLA as described in Methods. A fluorescence signal from each detected pair is seen as a distinct individual dot (red) whereas the nuclei are shown in blue. Images are magnifications from maximum intensity projections over the entire image volume, captured with a 20X objective on an AxioImager M2 fluorescence microscope. The Z-stacks were analyzed with Blob Finder software. N = 4. **b** PC3 cells were treated with 10 nM FVIIa for 1, 5, 15, and 20 min and the Mean Fluorescence Intensity (MFI) of activated ITGβ1 was monitored by flow cytometry as described in Methods. N = 5. **c** PC3 cells were incubated with scramble or ITGβ1 siRNA for 72 h and then treated with 100 nM FVIIa for 30 min. The phosphorylation of the IGF-1R was analyzed by PLA and antibodies toward IGF-1Rβ and phosphotyrosine (pY). N = 3–11. * = p ≤ 0.05 ** = p ≤ 0.01 *** = p ≤ 0.001

PC3 prostate cancer cells were treated with FVIIa for 30 min, and the proximity between TF and activated ITGβ1 on the cell surface was investigated by PLA in situ. Only a few signals were found in the untreated control cells, whereas the number of signals was increased about three-fold by FVIIa treatment (Fig. 1a). The PC3 cells were treated with FVIIa for shorter time periods, and the ITGβ1 activation was monitored by flow cytometry. A significant increase was detected after 5, 15, and 20 min (Fig. 1b). Next, the transactivation of IGF-1R by TF/FVIIa was evaluated by PLA in situ in PC3 cells (serving as control), and in PC3 cells treated with scramble and ITGβ1 siRNA (Fig. 1c). In both the control cells and the scramble-treated cells a two-fold increase of IGF-1R phosphorylation was detected. No activation of the IGF-1R could be recorded in the ITGβ1 siRNA treated cells. These experiments indicate that FVIIa treatment increases the interplay between ITGβ1 and TF and that the subsequent activation of ITGβ1 is important for the TF/FVIIa-dependent transactivation of IGF-1R.

### The FVIIa-induced phosphorylation of the IGF-1R is regulated by the caveolin-1 scaffolding domain.

**Fig. 2:**
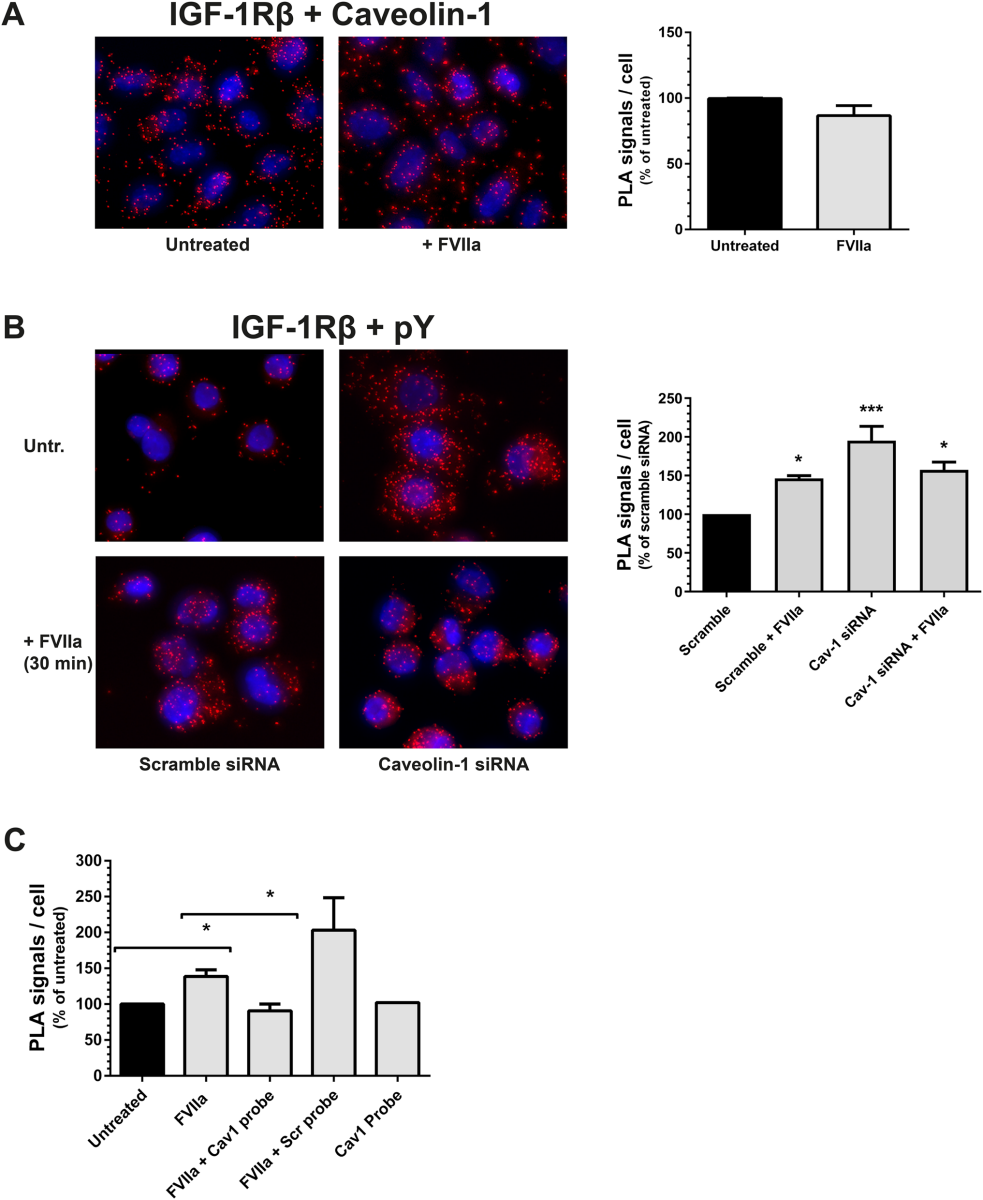
The FVIIa-induced phosphorylation of the IGF-1R is regulated by the caveolin-1 scaffolding domain **a** PC3 cells were treated with 100 nM FVIIa for 30 min, and the proximity of IGF-1Rβ and Cav1 was determined by PLA as described in Methods. A fluorescence signal from each detected pair is seen as a distinct individual dot (red) whereas the nuclei are shown in blue. Images are magnifications from maximum intensity projections over the entire image volume, captured with a 20X objective on an AxioImager M2 fluorescence microscope. The Z-stacks were analyzed with Blob Finder software N = 3. **b** PC3 cells were incubated with siRNA targeting Cav1 or a scrambled sequence for 72 h and then treated with 100 nM FVIIa for 30 min. The phosphorylation of the IGF-1R was assessed using PLA as described in Methods. Red = PLA signals, blue = nuclei. N = 3. **c** PC3 cells were preincubated for 3 h with 5 nM of either a Cav1 scaffolding domain peptide or a scrambled peptide of equal length, and then treated with 100 nM FVIIa for 30 min. The phosphorylation of the IGF-1R was recorded using PLA and quantified as described in Methods. N = 3. * = p ≤ 0.05 *** = p ≤ 0.001

The IGF-1R and Cav1 proteins were found to be in close proximity in situ in resting cells and the treatment with FVIIa did not alter the distance between the proteins in any way reflected by the PLA assay (Fig. 2a). Since Cav1 is the defining protein of caveolae, it is plausible to assume that the IGF-1R is present within caveolae on the PC3 cellular surface.

Cav1 expression was then decreased in the PC3 cells by siRNA which led to an increased phosphorylation of IGF-1R (Fig. 2b). FVIIa treatment induced IGF-1R phosphorylation in the scramble group but did not further contribute to IGF-1R activation in cells with reduced levels of Cav1 (Fig. 2b).

To further examine the role of Cav1, PC3 cells were pre-treated for 3 h with a commercially available, cell membrane penetrating Cav1 scaffolding domain peptide (mimicking Cav1 overexpression) or with a scrambled Cav1 scaffolding domain peptide and then incubated with FVIIa for 30 min (Fig. 2c). The IGF-1R phosphorylation was increased in FVIIa-treated cells and in the cells treated with FVIIa and scrambled peptide, but the TF/FVIIa-complex did not induce phosphorylation of IGF-1R in the Cav1 scaffolding domain peptide group. This suggests that Cav1 has a dampening effect on IGF-1R activation.

### Simvastatin treatment inhibits caveolin-1 transcription and increases the phosphorylation of the IGF-1R

**Fig. 3:**
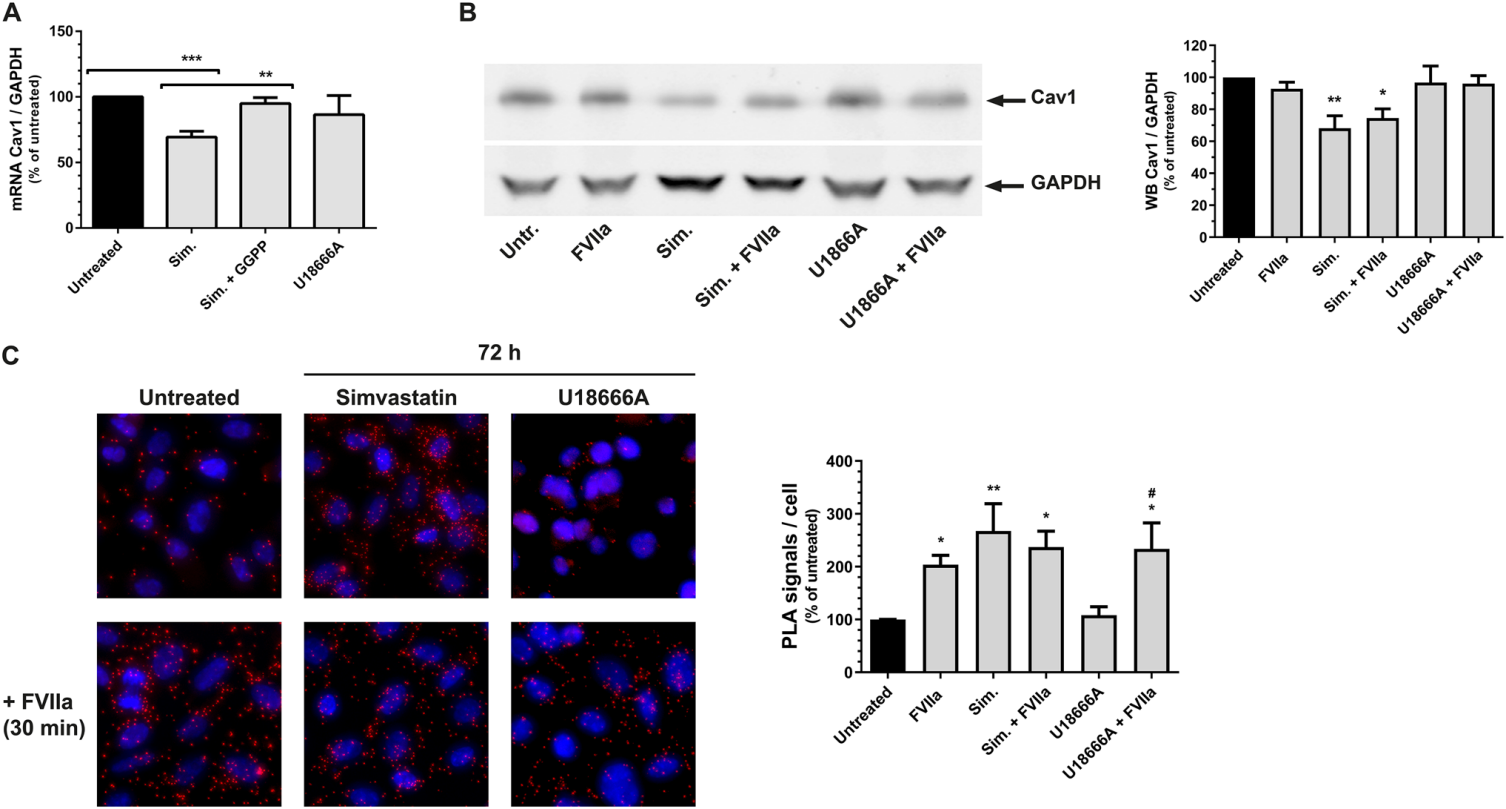
Simvastatin treatment increases the phosphorylation of the IGF-1R by down-regulation of caveolin-1 **a** PC3 cells were treated for 72 h with 500 nM simvastatin, 500 nM simvastatin + 5 µM GGPP, or with 1.25 µM U18666A. Cav 1 mRNA levels were then assessed using qRT-PCR as described in Methods and normalized toward GAPDH. N = 4–5. **b** PC3 cells were treated with 500 nM simvastatin or 1.25 µM U18666A for 72 h and then incubated with 100 nM FVIIa for 30 min. Cav1 protein levels were then assessed using WB as described in Methods and normalized toward GAPDH. N = 5. **c** PC3 cells were treated with 500 nM simvastatin or 1.25 µM U18666A for 72 h and then incubated with 100 nM FVIIa for 30 min. The phosphorylation of the IGF-1R was then assessed using PLA as described in Methods. Red = PLA signals, blue = nuclei. N = 6–8. * = p ≤ 0.05 ** = p ≤ 0.01 *** = p ≤ 0.001. # = p ≤ 0.05 vs U18666A treated cells

Data describing a reduction of Cav1 mRNA levels in canine cells after treatment with the cholesterol synthesis inhibitor simvastatin have previously been published [35]. PC3 cells were therefore incubated with or without different concentrations of simvastatin or the cholesterol transport inhibitor U18666A in normal culture media for different time-points. The distribution of cell surface cholesterol (S-Fig. 2) as well as TF and IGF-1R mRNA levels were monitored (S-Fig. 3). Based on these data, we decided to use 500 nM simvastatin, 1.25 µM U18666A and the time-point 72 h for further experiments. At these settings, the cell surface levels of cholesterol were reduced by both treatments but the transcription of TF and IGF-1R were unaffected.

The Cav1 expression was then measured after incubation with FVIIa, simvastatin and U18666A. Treatment with simvastatin, but not U18666A, reduced Cav1 mRNA and protein levels by approximately 30% (Fig. 3a and b) indicating that the cell surface level of cholesterol was not involved in the regulation of Cav1. Co-incubation of the PC3 cells with 500 nM simvastatin and the cholesterol synthesis pathway intermediate GGPP blunted the effects of simvastatin on Cav1 mRNA transcription (Fig. 3a). Simvastatin thus regulates the transcription of Cav1 in a seemingly cholesterol independent manner.

Addition of FVIIa to untreated cells, or to cells treated with U18666A increased the phosphorylation of the IGF-1R, whereas the IGF-1R was activated regardless of the presence of FVIIa in the simvastatin treated cells (Fig. 3c). U18666A had no effect on its own on the IGF-1R phosphorylation. Similar results were thereby recorded after reduction of Cav1 by simvastatin as after treatment with Cav1 siRNA.

### TF/FVIIa induces a phosphorylation of tyrosine 14 on caveolin-1 in a β1 integrin-dependent manner

**Fig. 4:**
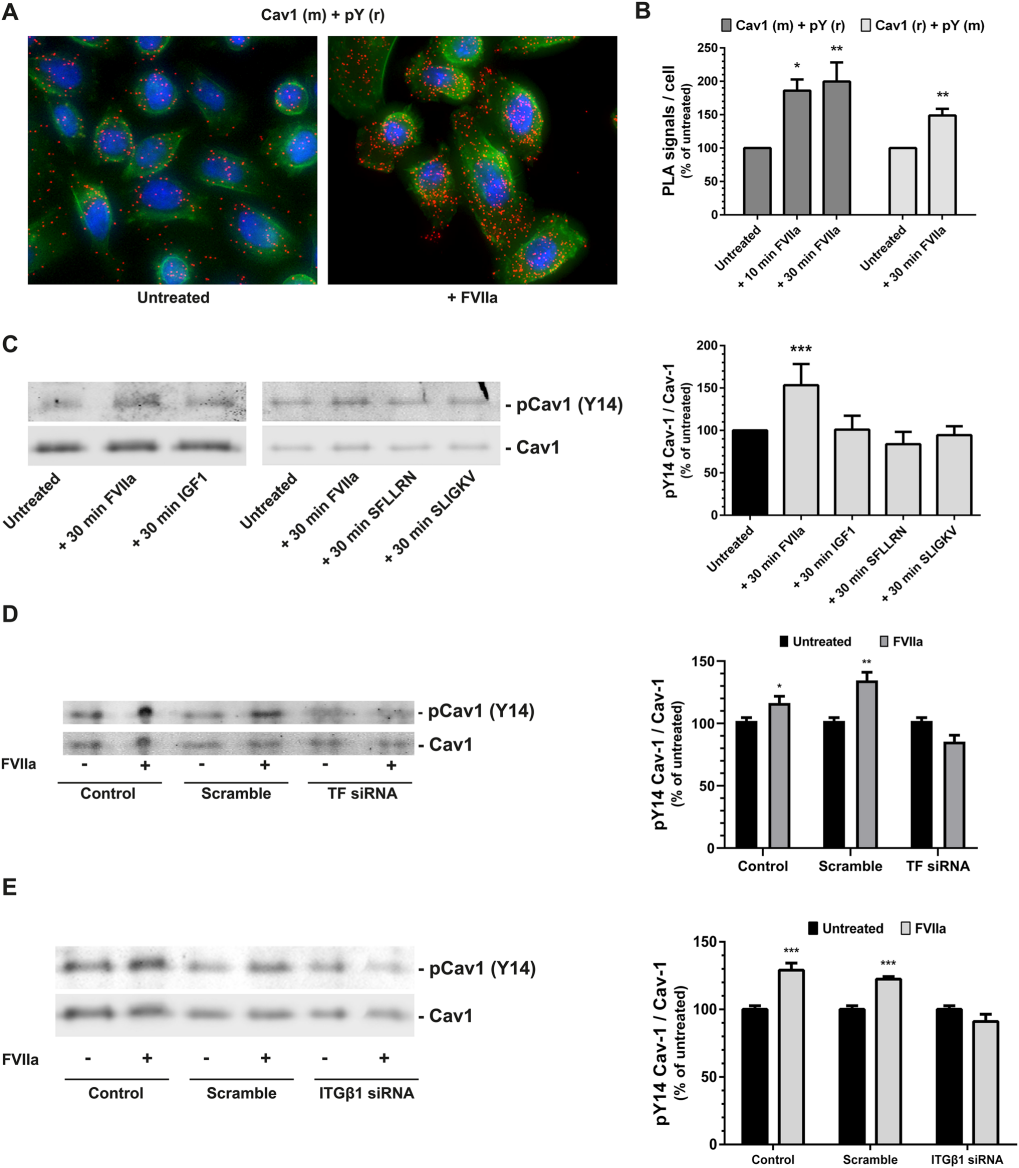
TF/FVIIa induces a phosphorylation of tyrosine 14 on caveolin-1 in a β integrin-dependent manner **a** PC3 cells were treated with 100 nM FVIIa for 10 min and the proximity of antibodies binding to Cav1 and phosphotyrosine (pY) was assessed using PLA as described in Methods. Red = PLA signals, blue = nuclei, green = cytoskeleton. Images are magnifications from maximum intensity projections over the entire image volume, captured with a 20X objective on an AxioImager M2 fluorescence microscope. The Z-stacks were analyzed with Blob Finder software. **b** The images captured in Fig. 4A as well as PLA images from PC3 cells treated with 100 nM FVIIa for 30 min were analyzed as described in Methods. Different combinations of mouse (m) and rabbit (r) antibodies binding to Cav1 and phosphotyrosine were used as indicated. N = 3–5. **c** PC3 cells were treated with 10 nM FVIIa, 100 ng/ml IGF1, 50 µl PAR1 agonist SFLLRN, or 50 µM PAR2 agonist SLIGKV for 30 min, and the pCav1 Tyr14 and Cav1 levels were analyzed by WB as described in Methods. The levels of pCav1 Tyr14 were normalized toward Cav1. N = 4–8. **d**-**e** PC3 cells were incubated with scramble, TF or ITGβ1 siRNA for 72 h and then treated with 10 nM FVIIa for 30 min. The pTyr14 phosphorylation of Cav1 was analyzed by WB as described in Methods. N = 4–6. * = p ≤ 0.05 ** = p ≤ 0.01 *** = p ≤ 0.001

Since FVIIa did not alter the proximity between Cav1 and IGF-1R (Fig. 2a) or the protein levels of Cav1 (Fig. 3b) we investigated if the TF/FVIIa-complex could induce Cav1 phosphorylation. The PC3 cells were treated with FVIIa and by utilizing PLA we recorded an increased pTyr of Cav1 after 10 and 30 min (Fig. 4a-b). We verified the PLA data by using a second Cav1—pTyr antibody pair with similar results (Fig. 4b).

An increase in Cav1 phosphorylation was also recorded by WB, using an antibody directed toward phosphorylated Cav1α, after 30 min of incubation with FVIIa (Fig. 4c). Treatment with recombinant IGF1 or the PAR1 agonist SFLLRN or the PAR2 agonist SLIGKV did not induce a Cav1 Tyr14 phosphorylation in our experimental setup (Fig. 4c, for functional testing of the PAR1 and PAR2 agonists please see S-Fig. 4). There was furthermore no phosphorylation of Cav1 by FVIIa after siRNA mediated down regulation of TF (Fig. 4d). This means that FVIIa needs to bind to TF to initiate the Cav1 phosphorylation.

To test if the TF/FVIIa-induced Cav1 phosphorylation was dependent on ITGβ1, PC3 cells were treated with ITGβ1 siRNA prior to the addition of FVIIa. In control cells and scramble siRNA-treated cells a Cav1 phosphorylation was recorded, whereas in the ITGβ1 knocked cells no Cav1 phosphorylation was detected (Fig. 4e). The combined data from the PLA and WB experiments thereby strongly indicate that FVIIa, in contrast to IGF1 and PAR1/2 agonists, phosphorylates the Cav1α isoform on tyrosine 14 in a TF and ITGβ1-dependent manner.

### The FVIIa-induced phosphorylations of caveolin-1 and IGF-1R are Src-family-dependent

**Fig. 5:**
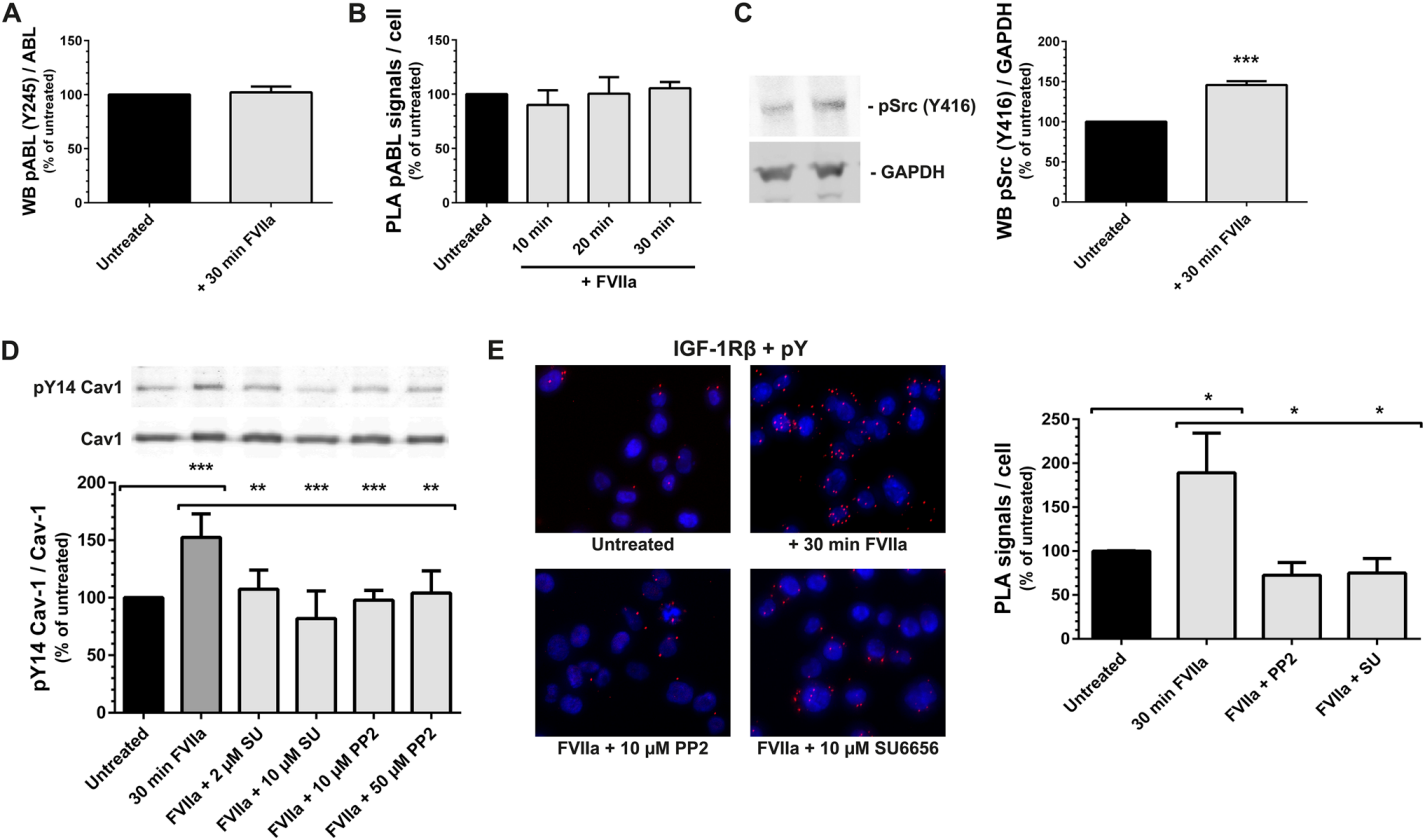
The FVIIa-induced phosphorylations of caveolin-1 and IGF-1R are Src-family dependent **a** PC3 cells were treated with 10 nM FVIIa for 30 min. Levels of pABL (Y245) and ABL were analyzed by WB as described in Methods and expressed as a ratio of pABL(Y245) / ABL. N = 4. **b** PC3 cells were treated with 100 nM FVIIa for 10, 20 and 30 min. The phosphorylation of the ABL was recorded using PLA and quantified as described in Methods. N = 2. **c** PC3 cells were treated with 10 nM FVIIa for 30 min. Protein levels of pSrc (Y416) and GAPDH were analyzed by WB as described in Methods and expressed as a ratio of pSrc (Y416) / GAPDH. N = 6. **d** PC3 cells were pretreated with the Src-family inhibitors SU6656 (SU) or PP2 for 1 h as indicated. The cells were then stimulated with 10 nM FVIIa for 30 min and the levels of pTyr14 on Cav1 assessed with WB as described in Methods. The immunoblots were quantified and pCav1 Tyr14 normalized toward Cav1. N = 4–8. **e** PC3 cells were pretreated with Src-family inhibitors for 1 h as indicated and then incubated with 100 nM FVIIa for 30 min. The phosphorylation of the IGF-1R was then assessed using PLA as described in methods. Red = PLA signals, blue = nuclei. N = 5–7. * = p ≤ 0.05 ** = p ≤ 0.01 *** = p ≤ 0.001

Cav1 Tyr14 is phosphorylated by kinases belonging to the Abl and Src families [36, 37]. We found no differences in Abl phosphorylation between untreated and FVIIa-treated PC3 cells when using an Abl Tyr245-specific antibody in WB (Fig. 5a). A higher concentration of FVIIa, different time points, and the use of the more sensitive PLA method produced similar results (Fig. 5b). In contrast, FVIIa induced a phosphorylation of Src family members on tyrosine 416 (Fig. 5c).

The PC3 cells were treated for one hour with two different concentrations of the Src inhibitors SU6656 or PP2 followed by incubation with FVIIa. Src inhibition reduced the FVIIa-induced phosphorylation of Cav1 to levels comparable to untreated cells (Fig. 5d). Similar results were also found in MDA-MB-231 breast cancer cells and DU145 prostate cancer cells (Fig. 8a and S-Fig. 5, respectively). The impact of Src inhibition on IGF-1R activation in FVIIa-treated PC3 cells was also determined in situ. The number of detectable pTyr IGF-1R PLA signals was reduced in the Src-inhibited PC3 cells (Fig. 5e). From these results we conclude that both the phosphorylation of Cav1 and the phosphorylation of IGF-1R by TF/FVIIa are sensitive to inhibition of Src-family kinases.

### The nuclear translocation of IGF-1R is dependent on β1 integrin and caveolin-1

**Fig. 6:**
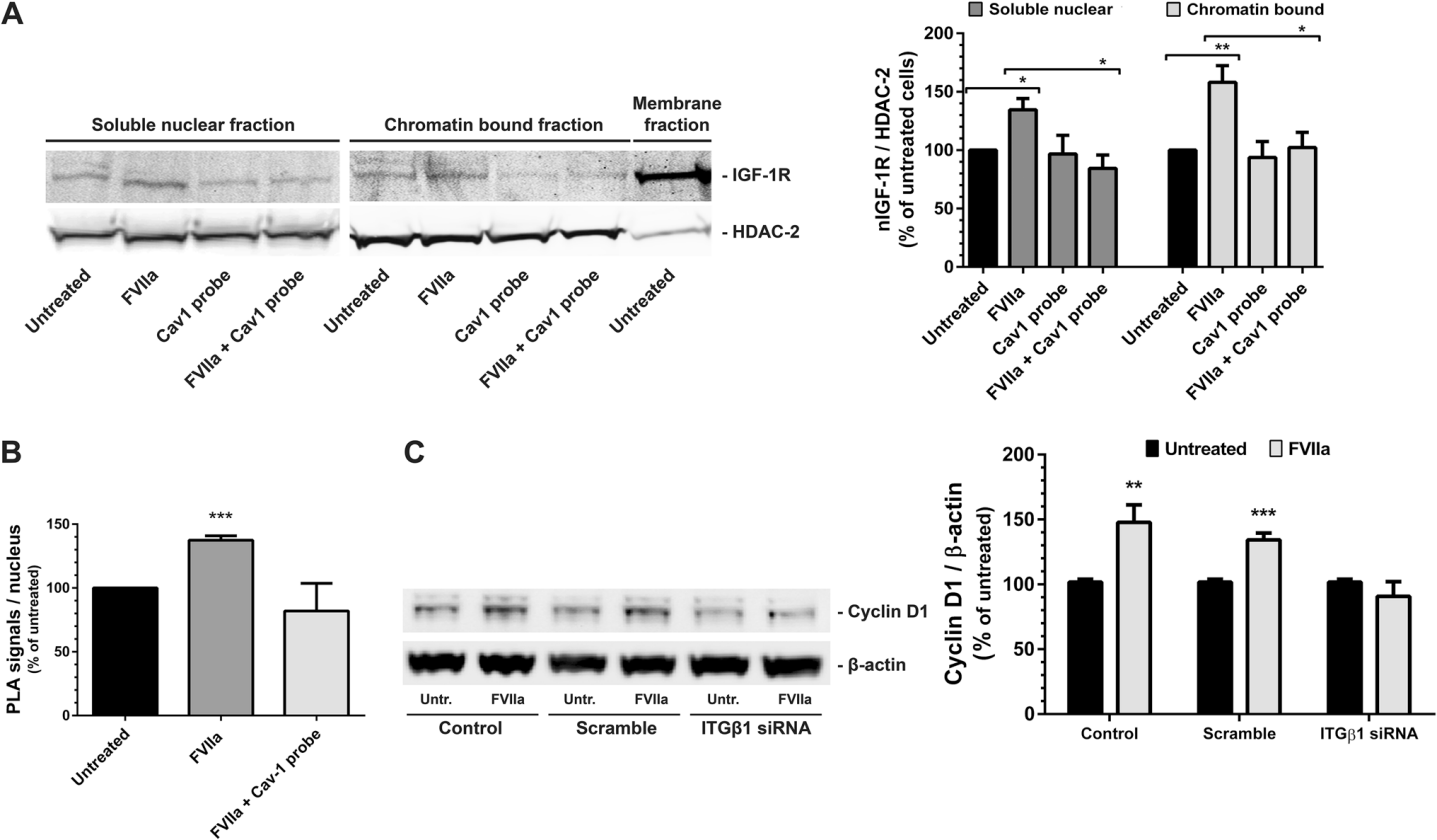
The nuclear translocation of IGF-1R is dependent on β1 integrin and caveolin-1 **a** PC3 cells were left untreated or were pre-incubated for 3 h with 5 nM of a Cav1 scaffolding domain peptide and were then stimulated with 10 nM FVIIa for 30 min. Membrane, nuclear soluble and chromatin-bound protein extracts were analyzed for IGF-1Rβ and HDAC-2 by WB as described in Methods. The nuclear soluble and chromatin-bound fractions were quantified and normalized toward HDAC-2. N = 4–5. **b** PC3 cells were left untreated or were pre-incubated for 3 h with 5 nM of a Cav1 scaffolding domain peptide and then stimulated with 100 nM FVIIa for 30 min. Nuclear IGF-1Rβ was detected using PLA and quantified as described in Methods. N = 3. **c** PC3 cells were incubated with scramble or ITGβ1 siRNA for 72 h and then treated with 10 nM FVIIa for 30 min. The protein levels of cyclin D1 and β-actin were analyzed by WB as described in Methods and expressed as a ratio of cyclin-D1 / β-actin. N = 5. * = p ≤ 0.05 ** = p ≤ 0.01 *** = p ≤ 0.001

PC3 cells were pre-treated with the Cav1 scaffolding domain peptide for 3 h (in the same concentration that prevented IGF-1R phosphorylation, Fig. 2c) before addition of FVIIa. The nuclear IGF-1R translocation was then assessed by WB on fractionated cell lysates. Nuclear IGF-1R was increased in both the soluble nuclear and chromatin-bound cell fractions post FVIIa treatment (Fig. 6a). The presence of the Cav1 scaffolding domain peptide completely blocked the FVIIa-dependent nuclear IGF-1R enrichment. Similar data was found by PLA in situ (Fig. 6b).

We have previously shown how TF/FVIIa signaling induce transcription of the cyclin-D1 gene in breast cancer cells by using real-time PCR [24]. Figure 6c shows that the induction of PC3 cells by FVIIa increased the cyclin-D1 protein levels in control cells and in scramble siRNA-treated cells, whereas ITGβ1 siRNA treatment blocked this up-regulation.

### The anti-apoptotic signaling by TF/FVIIa/IGF-1R is dependent on β1 integrin and caveolin-1

**Fig. 7:**
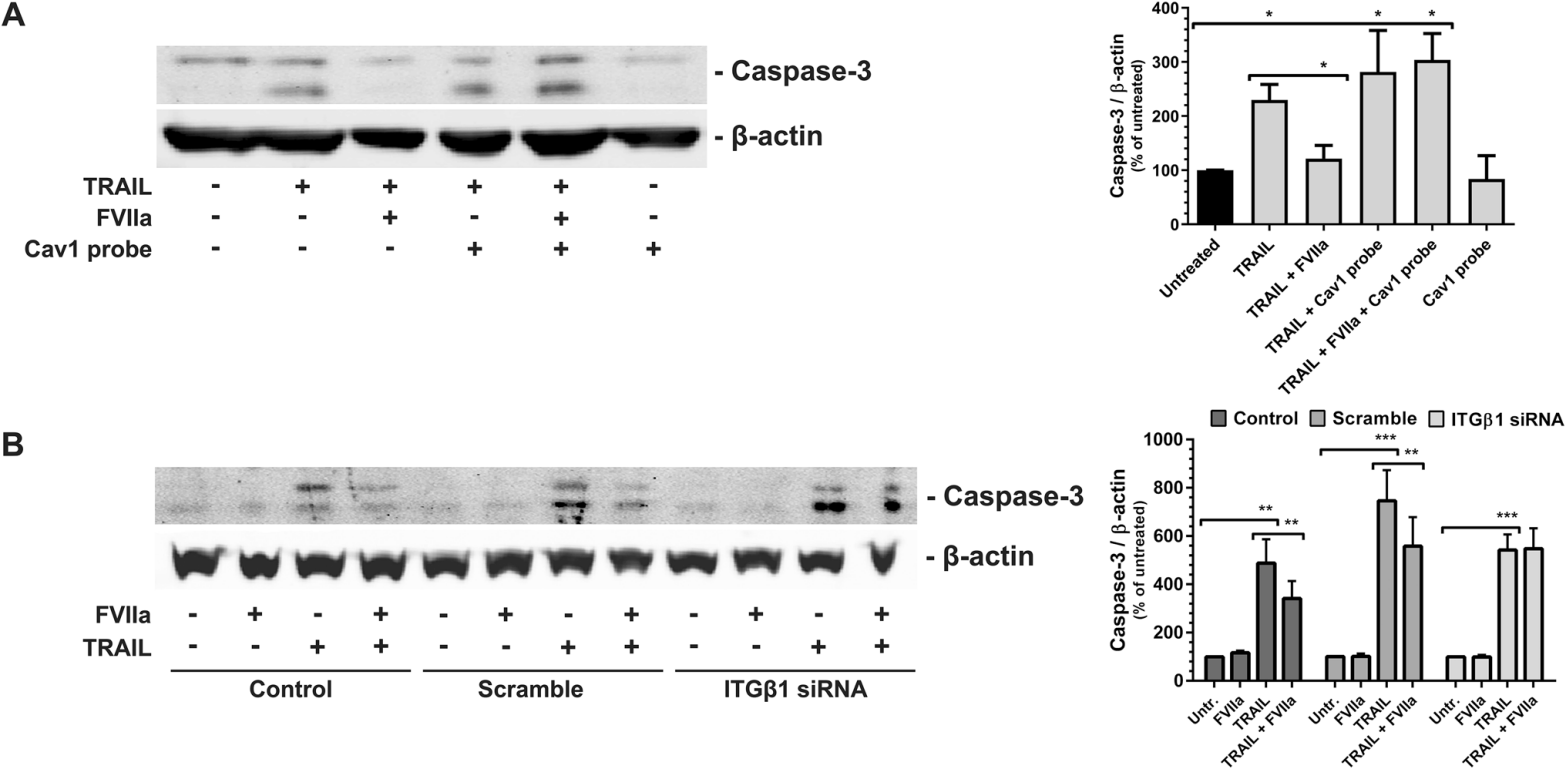
The anti-apoptotic signaling by TF/FVIIa/IGF-1R is dependent on β1-integrin and caveolin-1 **a** PC3 cells were treated with 100 ng/ml TRAIL, 5 nM Cav1 scaffolding domain peptide, and 10 nM FVIIa for 3 h as indicated. Apoptosis was monitored by measuring the activation of caspase-3 with WB as described in Methods. The caspase-3 bands were quantified and normalized toward β-actin. N = 3–4. **b** PC3 cells were incubated with scramble or ITGβ1 siRNA for 72 h and then treated with 100 ng/ml TRAIL and 10 nM FVIIa for 3 h as indicated. Apoptosis was monitored by measuring the activation of caspase-3 with WB as described in Methods. The caspase-3 bands were quantified and normalized toward β-actin. N = 6–8. * = p ≤ 0.05 ** = p ≤ 0.01 *** = p ≤ 0.001

Apoptosis was induced in the PC3 cells by treatment with the death receptor agonist TRAIL for 3 h. The cells were also treated with the Cav1 scaffolding-domain peptide and/or FVIIa and the activation of caspase-3 was monitored by WB (Fig. 7a). The activation of caspase-3 by TRAIL was reduced in the presence of FVIIa but the protective effect of FVIIa was canceled by the Cav1 peptide. The Cav1 peptide did not activate caspase-3 on its own.

The PC3 cells were then treated with siRNA towards ITGβ1 and again challenged by TRAIL (Fig. 7b). FVIIa retained its protective effect in the control cells and the cells transfected with scramble siRNA, whereas the knockdown of ITGβ1 resulted in a loss of anti-apoptotic signaling by the TF/FVIIa complex.

**Fig. 8:**
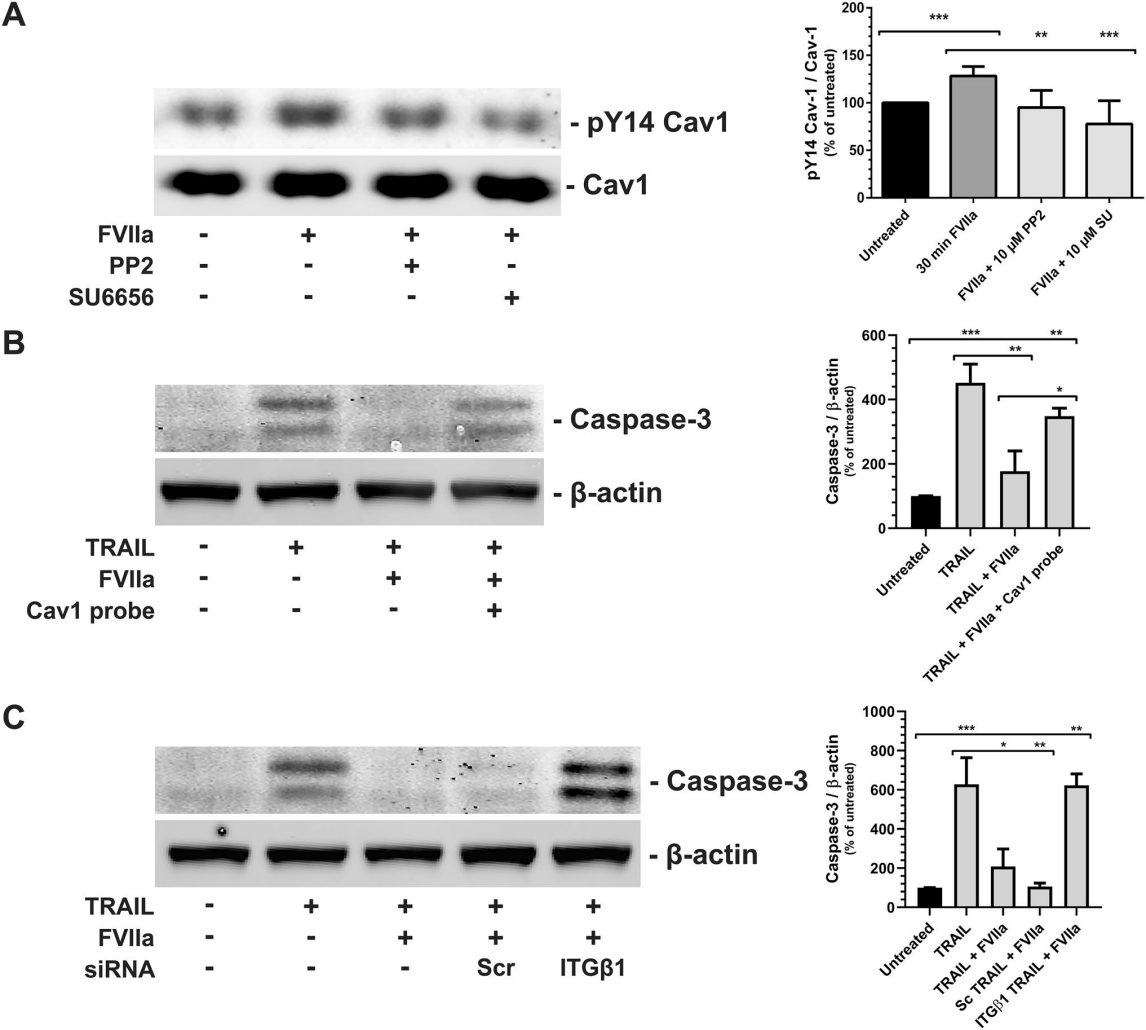
Caveolin-1 phosphorylation and anti-apoptotic signaling in MDA-MB-231 cells after FVIIa treatment. **a** MDA-MB-231 cells were pretreated with the Src-family inhibitors SU6656 (SU) or PP2 for 1 h as indicated. The cells were then stimulated with 10 nM FVIIa for 30 min and the levels of pTyr14 on Cav1 assessed with WB as described in Methods. The immunoblots were quantified and pCav1 Tyr14 normalized toward Cav1. N = 6–7. **b** MDA-MB-231 cells were treated with 100 ng/ml TRAIL, 5 nM Cav1 scaffolding domain peptide, and 10 nM FVIIa for 3 h as indicated. Apoptosis was monitored by measuring the activation of caspase-3 with WB as described in Methods. The caspase-3 bands were quantified and normalized toward β-actin. N = 5–6. **c** PC3 cells were incubated with scramble or ITGβ1 siRNA for 72 h and then treated with 100 ng/ml TRAIL and 10 nM FVIIa for 3 h as indicated. Apoptosis was monitored by measuring the activation of caspase-3 with WB as described in Methods. The caspase-3 bands were quantified and normalized toward β-actin. N = 4. * = p ≤ 0.05 ** = p ≤ 0.01 *** = p ≤ 0.001

Finally, we successfully verified the FVIIa-dependent Cav1 phosphorylation (Fig. 8a) and the importance of Cav1 (Fig. 8b) and ITGβ1 (Fig. 8c) on the anti-apoptotic signaling downstream of TF in MDA-MB-231 breast cancer cells.

## Discussion

**Fig. 9:**
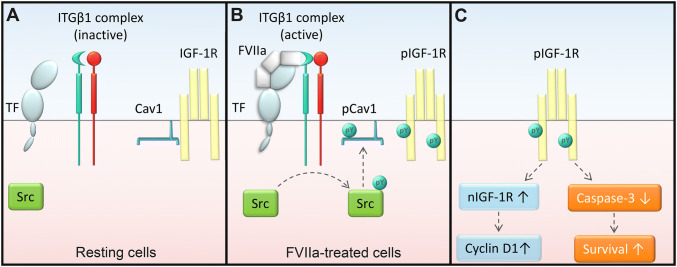
The anti-apoptotic signaling by TF/FVIIa/IGF-1R is dependent on β1-integrin and caveolin-1. The proposed mechanism is summarized as follows **a** In resting cells, the ITGβ1 complex is largely in an inactive state and caveolin-1 is associated with one or both β-chains of the membrane bound IGF-1R through the Cav1 scaffolding domain. **b** The ITGβ1 complex is activated by interaction with FVIIa after formation of the TF/FVIIa complex. Activated ITGβ1 induces phosphorylation of Src family kinases which in turn phosphorylates Cav1. This terminates the inhibition of IGF-1R by Cav1 and IGF-1R is tyrosine phosphorylated. **c** The activation IGF-1R leads to an increased resistance to TRAIL-induced apoptosis. The receptor is also translocated to the nucleus where it binds to DNA and induces an increase in cyclin D1 levels

The aim of this study was to determine the pathway between TF and the RTK IGF-1R after FVIIa treatment, with the subsequent anti-apoptotic effect and protein production as biological read-outs. We propose a model that starts with TF/FVIIa-dependent activation of ITGβ1, which leads to Src-dependent phosphorylation of Cav1 and finally IGF-1R signaling and downstream effects (Fig. 9).

Signaling by the TF/FVIIa complex is known to induce transactivation of the EGFR and PDGFRβ. The signaling pathways between TF and these two RTKs have been described as Src-family dependent [38, 39] and similar data can now be shown for IGF-1R, with the addition that phosphorylation of Src was found to be conveyed by ITGβ1. Integrins are activated by conformational changes of the extracellular domains to induce bi-directional signaling of importance for e.g. cell survival [40, 41]. An interplay between TF and β1, α3, and α6 integrin subunits has been reported by others [14]. Consistent with our previously published data on the transactivation of IGF-1R by TF/FVIIa [18, 24] is the formation of the TF/FVIIa/ITGβ1 complex dependent on FVIIa but not on a phosphorylated cytoplasmic TF domain or the activation of PAR2 [15]. In our experimental setup, extracellular matrix (ECM) from serum in the cell media or produced by the cells might have contributed to the transactivation of IGF-1R by interacting with ITGβ1. Both long and short effects in our experiments were, however, seen after the addition of FVIIa to the cells. This indicates that ITGβ1 expression per se is insufficient. FVIIa treatment increases the amount of ITGβ1 present in its active conformation, which indicates that regardless binding of ITGβ1 to ECM or not, there is a dependency on the formation of the TF/FVIIa-complex for this activation.

Interestingly, an integrin-binding motif in the FVIIa protease domain required for the association of TF/FVIIa with ITGβ1 was recently described [15]. This means that TF, in this case, most likely functions as a co-factor/scaffold to FVIIa which in turn associates with ITGβ1 to induce the conformational changes required for ITGβ1 activation. In the light of this information should the PLA data presented in Fig. 1a therefore be interpreted as a complex formation between TF/FVIIa/activated ITGβ1 and not only between TF and activated ITGβ1.

Two antibodies directed at different domains of TF affect the interaction of the binary TF-complex and ITGβ1 in opposite ways. The antibody TF-5G9 reportedly increases the association of TF with integrins on a similar level as FVIIa, whereas TF-10H10 specifically disrupts this association [14]. We have previously published data that shows a negative impact of TF-10H10 on the IGF-1R dependent anti-apoptotic effects of FVIIa [18]. This fits with the assumption that the protective effect of TF/FVIIa is ITGβ1-dependent.

Many proteins regulated by caveolin, including IGF-1R, share a common amino acid sequence motif that binds to the Cav1 scaffolding domain [30]. This interaction inhibits the autophosphorylations of EGFR in A431 cells and CHO cells and of PDGFα and -β in NIH3T3 fibroblasts [42, 43]. It also stabilizes TGFβR1, and TrkA in inactive conformations until activation by ligands [44, 45]. Our data suggest that the scaffolding domain seems to have an impeding effect also on IGF-1R activation. The Cav1 scaffolding domain peptide used in this study have previously been shown to block eNOS activity and cellular NO release in vitro and to reduce inflammation and tumorigenesis in vivo, but data on its role in IGF-1R signaling is lacking [31, 46].

There are contrasting results presented in the literature concerning the mutual relations of IGF1 signaling and Cav1. Knockdown of Cav1 reduces e.g. IGF1 signaling in H9C2 rat cardiomyoblasts [47] whereas Cav1 overexpression significantly increases IGF1-induced internalization of IGF-1R in human hepatocellular carcinoma cells [48]. Cav1 is also Tyr14 phosphorylated in response to IGF1 treatment in mouse fibroblasts transfected with human IGF-1R [49]. Some of these discrepancies might be explained by the biological differences between a direct agonist activation of a receptor and a receptor transactivation. The transactivation of IGF-1R and PDGFRβ by TF/FVIIa leads to a delayed, partial activation of the receptor [24, 39]. Similarly, integrin-mediated activation of Src in urinary bladder carcinoma cells leads to a phosphorylation pattern in the kinase domain of EGFR that is different from that induced by the binding of EGF to EGFR [50]. The kinetics of Cav1 phosphorylation seems also to depend on the received stimuli. The activation after IGF1 treatment is recordable between 5 and 15 min in mouse fibroblasts [49] but not after 30 min. In contrast, it is still present at 30 min after addition of FVIIa in our model system. Considering these differences, it is difficult to discuss the conflicting data on a detailed level except to draw the conclusions that the outcome of protein interactions with Cav1 seems, to some extent, to be dependent on the cell type, the proteins, and type of stimuli. Since we were able to show a TF dependent phosphorylation of Cav1 by FVIIa (Fig. 4d) in three different cell lines (Fig. 5d, Fig. 8a, S-Fig. 5) this connection was, however, not exclusive to PC3 cells.

In the present study we focused on finding a mechanism to explain the transactivation of IGF-1R by TF/FVIIa and the previously known biological mechanisms of increased cell survival and IGF-1R nuclear translocation. The wide-spanned effects of IGF-1R and others RTKs entail a tight regulation of transcription and translation and also of their ligand availability. Since RTK activity drives many types of diseases, much effort has been put into the development and testing of pharmacological inhibitors towards the different receptors. The spatial distribution of receptors and the bypassing of ligands could, however, substantially affect the success of such targeted therapies, which is exemplified by the apparent failures of IGF-1R inhibitors in cancer patients [22, 51]. RTK transactivation and the aberrant spatial regulation of RTKs are therefore regarded as a promising field in the search for new therapies [52].

The first report on TF-dependent anti-apoptotic signaling described a protective effect of FVIIa in serum starved baby hamster kidney cells [17]. In a later study we detected activated caspase-8 24 h after serum withdrawal in breast cancer cells and noticed that treatment with FVIIa reduced the formation of the death inducing signaling complex (DISC) responsible for caspase-8 activation [18]. These data provided a new angle on TF/FVIIa-mediated cell survival aiming at the extrinsic pathway. To get a more refined cellular model we have since then treated cancer cells with TRAIL which triggers apoptosis by binding to DR4/DR5 receptors. This binding leads to intracellular assembly of the DISC and cleavage of caspase-8 which then directly activates caspase-3 i.e.the apoptosis marker used in this study. In vivo, TRAIL is expressed in many tissues and on the surface of some immune cells [20, 21] and is present both as a cell bound protein and in a soluble form. For instance, TRAIL-expressing T cells are described to induce apoptosis in vascular smooth muscle cells (VSMC) after cell–cell interactions whereas IFNα-treated monocytes are primed to release the soluble form of TRAIL [21, 53]. Recombinant TRAIL has gained interest as a potential cancer therapeutic drug and the power of TRAIL-based therapies stems from TRAILs explicit cancer cell-selectivity over normal cells. Recent reports do, however, report that tumor cells can acquire TRAIL resistance by several mechanisms [19]. VSMC and many cancer cells express TF and the presence of FVIIa within the plaque or in the vicinity of the tumor may therefore result in both a decreased risk for plaque rupture and an increased survival of the cancer cells. The TF/FVIIa-dependent anti-apoptotic effects may thereby have a dual role in the body. This is of importance to keep in mind since the TF-10H10 antibody and the Cav1 scaffolding domain peptide both target the TF/FVIIa/IGF-1R pathway and might thus serve as treatment tools in different diseases. As a cautionary note, recent publications assign TF-dependent signaling pro-apoptotic properties [54, 55]. When and why TF favors one way over the other is today not known. The pro-apoptotic signaling has, however, been recorded in non-malignant cells after MAP kinase activation, i.e. different signaling pathways to the one described in this paper.

In summary, our data provide a plausible explanation of the interaction between the coagulation and the IGF-1R systems, which hopefully may contribute to explain the pathology in diseases associated with IGF-1R overexpression and increase the knowledge of TF-mediated anti-apoptotic signaling.

## Electronic supplementary material

Below is the link to the electronic supplementary material.

Supplementary file1 (DOCX 2323 kb)
