# Supplementary material for: Activation of β1 integrins and caveolin-1 by TF/FVIIa promotes IGF-1R signaling and cell survival
Source: Apoptosis. 2020 May 27;25(7):519–34. doi: 10.1007/s10495-020-01611-7 (PMC7347522; doi:10.1007/s10495-020-01611-7)
Supplement: Supplementary file 1 — Supplementary file1 (DOCX 2323 kb) [file 10495_2020_1611_MOESM1_ESM.docx]

## Supplemental data

**S-fig 1. siRNA**





A) Silencer® Select Validated siRNA toward caveolin-1 (Cav1), integrin β1 (ITGβ1), tissue factor (TF), and scramble RNA (10 nM, Assay-on-demand, Applied Biosystems, USA), were transfected into A) PC3 cells or B) MDA-MB-231 cells (ITGβ1 only) using Lipofectamine® RNAiMAX Transfection Reagent (Life Technologies). The protein levels of targeted proteins and IGF-1R were analyzed 72 h post transfection by western blot as indicated. Briefly, the cells were lyzed in 2% SDS sample buffer supplemented with 5% beta-mercaptoethanol. The proteins were then separated by SDS-PAGE and transferred onto Immobilon-FL PVDF membranes (Merck Millipore). The membranes were blocked in Odyssey blocking buffer (Licor) and left overnight at 4°C in blocking buffer containing the primary antibodies. The membranes were then washed in TBS 0.01% Tween-20 and incubated with secondary antibodies conjugated to IR-Dyes 680 and 800 for 60 minutes (Licor). The membranes were scanned and the bound proteins were visualized with the Odyssey Infrared Imaging System (Licor) and quantified using Odyssey V3.0 software. Representative blots are shown.

**S-fig 2: Cholesterol measurements in PC3 cells**

**
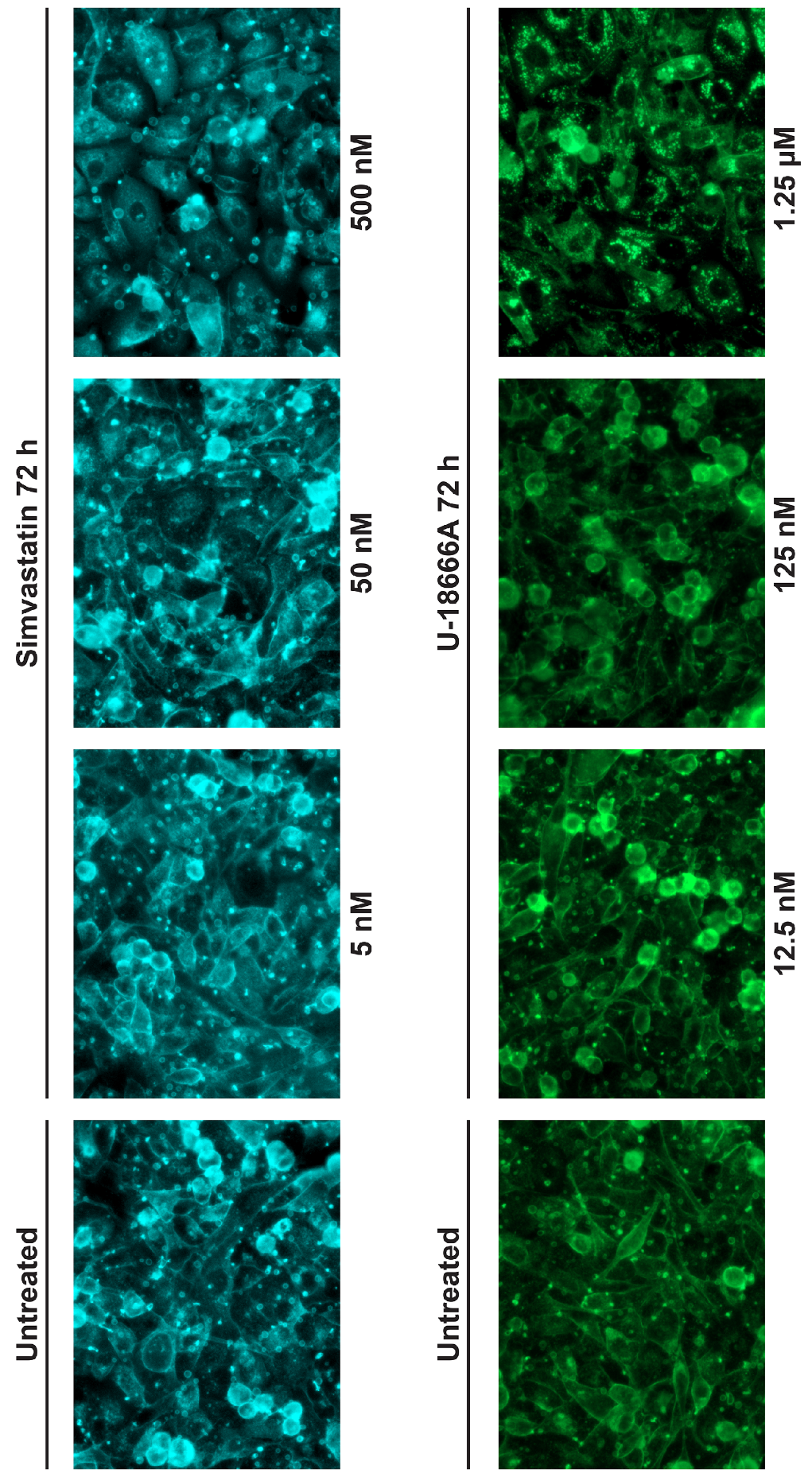
**

PC3 cells were treated as indicated and then stained for cholesterol using a Cholesterol Cell-Based Detection Assay Kit (Cayman Chemical, USA). In this kit, the ability of Filipin III to form a complex with cholesterol is utilized. This interaction alters the filipin absorption and fluorescence spectra allowing the complexes to be captured with a microscope (Zeiss Axiovert 40 CFL). Surface cholesterol was seen as thin glowing edges lining the cells. Increasing the concentration of simvastatin reduced the surface cholesterol, leaving an indistinct cytoplasmic staining. Treatment with the cholesterol transport inhibitor U18666A retained the cholesterol in a dose-dependent manner. The cholesterol is seen as small, green dots in the cytoplasm.

**S-fig 3: No induction of tissue factor or IGF-1R mRNA after treatment with simvastatin or U18666A**


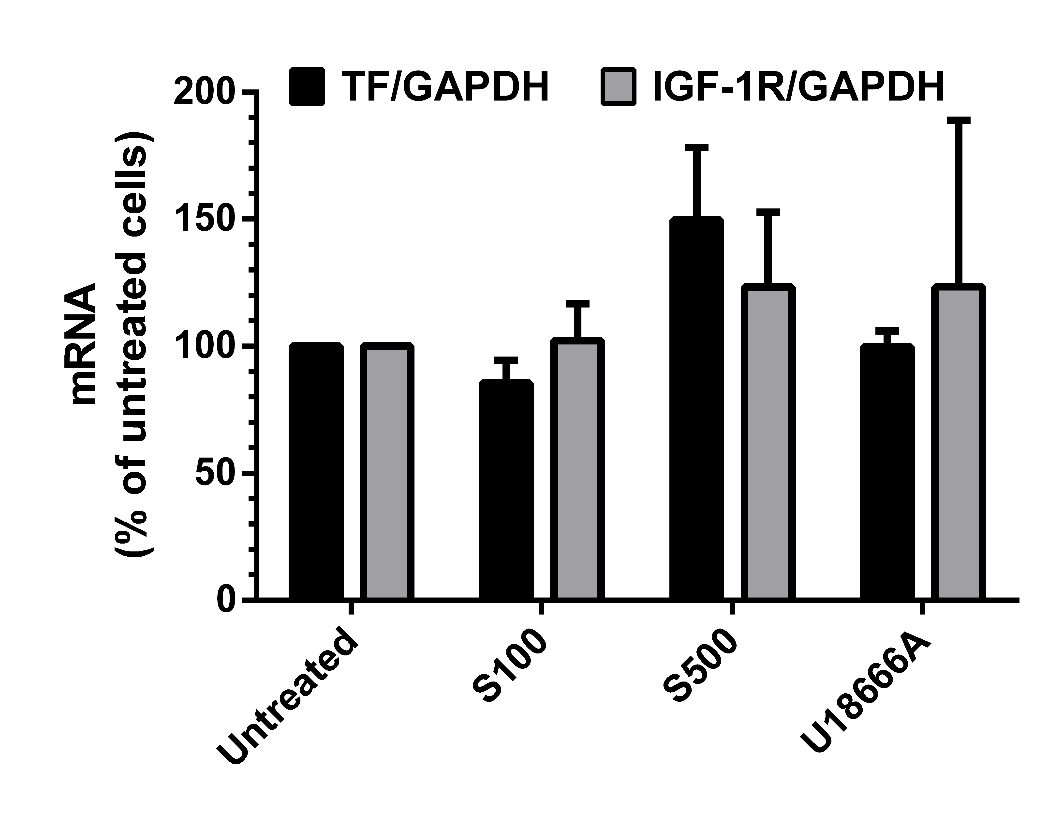


PC3 cells were treated with 100 or 500 nM simvastatin or 1.25 µM of the cholesterol transport inhibitor U18666A for 72 h. Real-time quantitative PCR analyses of tissue factor, IGF-1R, and GAPDH (Assay-on-demand, Applied Biosystems, USA) were performed on cDNA originating from oligoDT (Invitrogen, USA) converted total RNA extracted using Trizol® (Invitrogen). The samples were run and analyzed on an AbiPrism 7500 system (Applied Biosystems). N = 2-4

**S-fig 4: PAR1 and PAR2 agonists induce IL-8 mRNA production in PC3 cells**

**
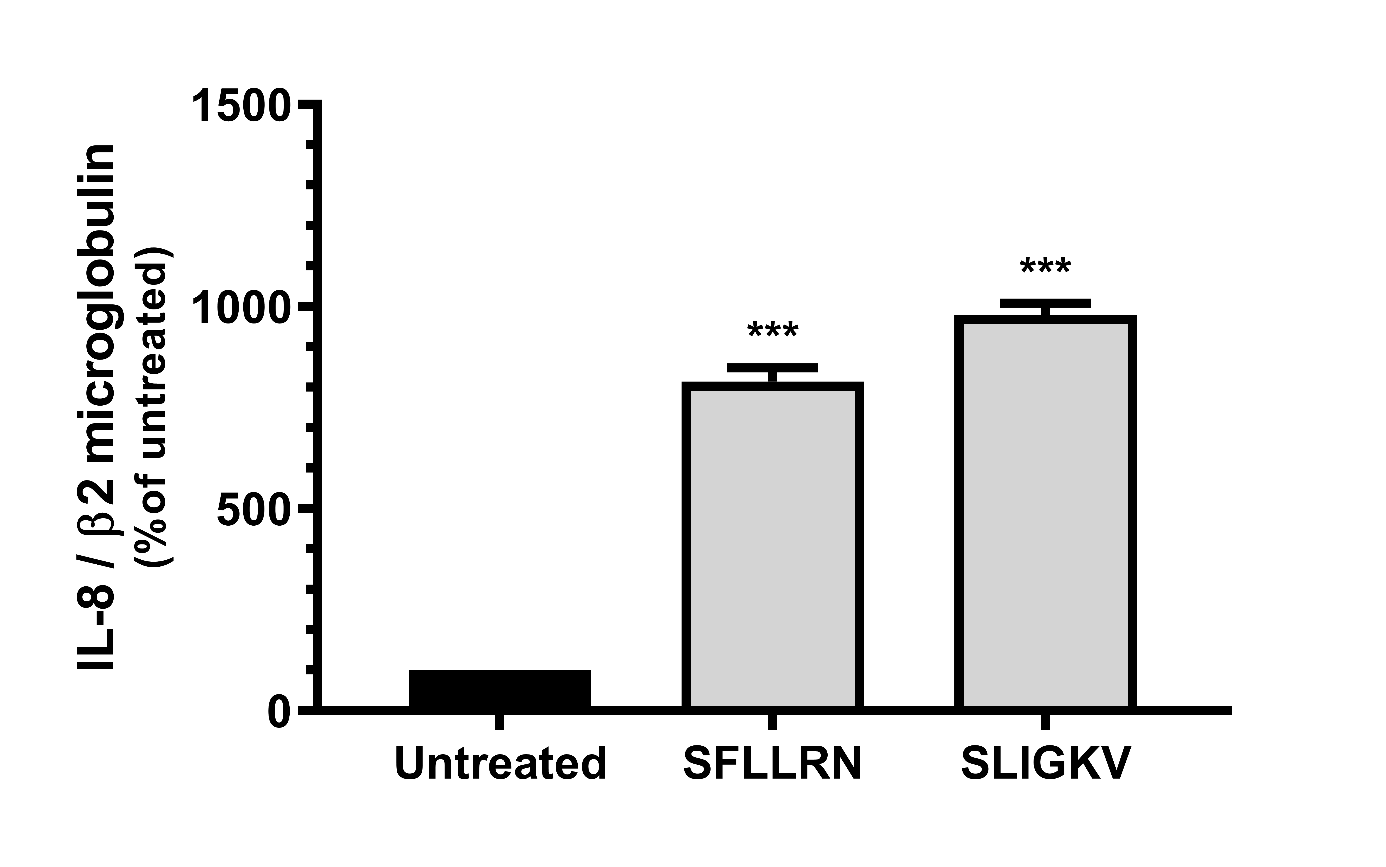
**

PC3 cells were treated with PAR1 (SFLLRN) or PAR2 (SLIGKV) agonists for one hour. Real-time quantitative PCR analyses of IL-8 and β2 microglobulin were performed on cDNA originating from oligoDT (Invitrogen, USA) converted total RNA extracted using RNeasy Mini Kit (Qiagen). The samples were run and analyzed on an AbiPrism 7500 system (Applied Biosystems). N = 3. *** = p≤0.001 vs untreated

**S-Fig 5.** **Phosphorylation of caveolin-1 by FVIIa stimulation is abolished in DU145 cells treated with Src-inhibitors.**

**

**

DU145 prostate cancer cells (N = 5-6) were pretreated with the Src-family inhibitors SU6656 (SU) or PP2 for 1 hour as indicated. The cells were then stimulated with 10 nM FVIIa for 30 minutes and the levels of pTyr14 on Cav1 assessed by WB. Briefly, the cells were lyzed in 2% SDS sample buffer supplemented with 5% beta-mercaptoethanol. The proteins were then separated by SDS-PAGE and transferred onto Immobilon-FL PVDF membranes (Merck Millipore). The membranes were blocked in Odyssey blocking buffer (Licor) and left overnight at 4°C in blocking buffer containing the primary antibodies. The membranes were then washed in TBS 0.01% Tween-20 and incubated with secondary antibodies conjugated to IR-Dyes 680 and 800 for 60 minutes (Licor). The membranes were finally scanned and the bound proteins were visualized with the Odyssey Infrared Imaging System (Licor) and quantified using Odyssey V3.0 software. pCav1 Tyr14 was normalized toward Cav1. * = p≤0.05 *** = p≤0.001.
